# A Case of Gorlin-Goltz Syndrome Presented with Psychiatric Features

**DOI:** 10.1155/2014/830874

**Published:** 2014-03-10

**Authors:** Amir Mufaddel, Mouza AlSabousi, Badr Salih, Ghanem AlHassani, Ossama T. Osman

**Affiliations:** ^1^Community Mental Health Services, Behavioral Sciences Institute, Al Ain Hospital, P.O. Box 1006, Al Ain, United Arab Emirates; ^2^Behavioral Sciences Institute, Al Ain Hospital, Al Ain, United Arab Emirates; ^3^College of Medicine and Health Sciences, United Arab Emirates University, P.O. Box 17666, Al Ain, United Arab Emirates

## Abstract

We report a case of a 34-year-old male who presented with an acute onset of pleomorphic psychiatric features. Upon examination we later diagnosed him with Gorlin-Goltz syndrome based on clinical and radiological findings that are characteristic for this rare autosomal dominant syndrome. His psychiatric manifestations included irritability, aggressive behavior, labile mood, hallucinations, paranoid delusions, and transient cognitive impairment. His past history indicated surgical excision of pigmented lesion in the left lower eyelid which turned out to be a basal cell carcinoma. His past visits to dermatology clinics indicated pitted keratosis involving hands, callosities, and seborrheic dermatitis. There were numerous palmar pits, and Brain CT Head scan revealed extensive calcification along falx cerebri and around the cerebellar vermis. He had low (20 ng/L) vitamin D level and high parathyroid hormone level. The patient improved using antipsychotic medications and vitamin D supplementations for symptomatic management and was discharged with a plan for multispecialty outpatient follow-up. This case highlights the importance of considering rare organic etiologies in the differential diagnosis of patients presenting with psychiatric symptoms. This is of vital importance for early intervention to prevent complications and for better outcomes of the coexistent diseases.

## 1. Introduction

Gorlin-Goltz syndrome, also known as nevoid basal cell carcinoma syndrome, is an autosomal dominant syndrome characterized by multiple clinical features involving the skin, nervous system, eyes, endocrine system, and bones [1]. The first report of this syndrome was made in 1894 by Jarisch and White who reported a patient with multiple basal cell carcinomas, scoliosis, and learning disability. The relationship between basal cell epitheliomas and developmental malformations was suggested by Binkley and Johnson in 1951 and Howell and Caro in 1959 [2]. Gorlin and Goltz, in 1960 described the classical triad of nevoid basal cell carcinoma including multiple basal cell carcinomas, keratocystic odontogenic tumors, in the jaws and bifid ribs that characterize the diagnosis of Gorlin-Goltz syndrome. Other established clinical features of this syndrome include calcification of the falx cerebri, palmar and plantar epidermal pits, spine and rib anomalies, relative macrocephaly, facial milia, frontal bossing, ocular malformation, meduloblastomas, cleft lip and/or palate, and developmental malformations [3, 4]. The intracranial calcifications may have no clinical significance or they may be critical in diagnosing the underlying pathology. Calcifications can be physiologic, dystrophic congenital, or vascular. They can also be due to congenital infections such as TORCH infections (toxoplasmosis, other [T. pallidum, Varicella-Zoster virus, Parvovirus B19], Rubellavirus Cytomegalovirus and Herpes Simplex Virus), acquired infections, and inflammatory lesions such as sarcoidosis and tumors. Metabolic disorders affecting calcium homeostasis predominantly involve the basal ganglia. Intracranial calcifications can also occur in rare idiopathic disorders such as Fahr disease [5]. The role of calcium in psychosis, agitation, and mania has been reported in the literature [6]. Carman and colleagues have reported small but statistically significant increases in serum total calcium and serum inorganic phosphorus associated with repeated onsets of psychotic agitation and manic symptoms [7].

## 2. Case Report

**Figure 1:**
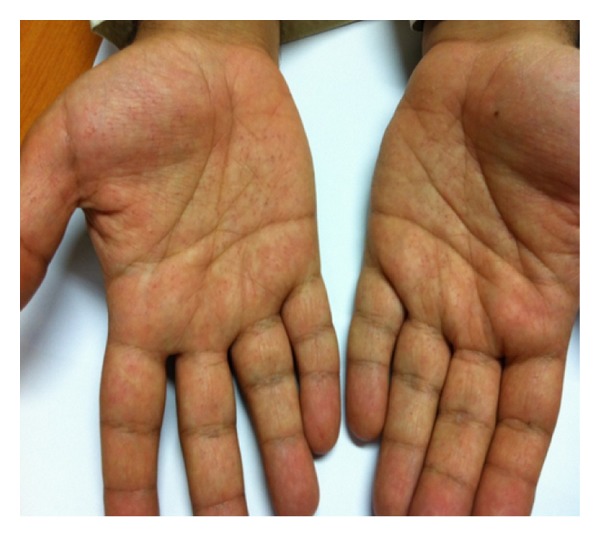
Numerous palmar pits in a patient with Gorlin-Goltz syndrome.

**Figure 2:**
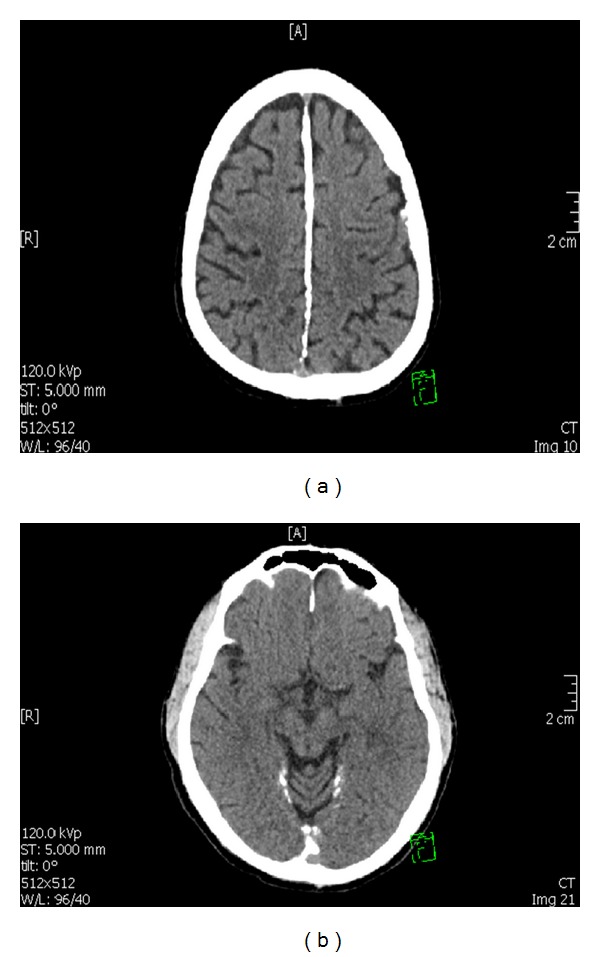
An axial brain CT scan showing extensive brain calcifications along falx cerebri (a) and around the cerebellar vermis with few tiny calcifications in the cerebellum bilaterally (b).

We report a case of a 34-year-old male presented to emergency department with acute onset of irritability and aggressive behavior. He was admitted to the psychiatric ward and was observed to have fluctuations in his symptoms. The patient reported that he ingested some glasses and plastic particles when he went mad which caused abdominal pain. Surgical opinion and abdominal ultrasound revealed no evidence of foreign body or any other abnormal finding. He reported a past episode of psychiatric symptoms in 1994 which lasted for 3 weeks and required hospital admission, but the patient was unable to recall details of his past symptoms. During his current hospitalization his mood was observed to be labile and he described his symptoms as if it was “a dream state or a movie.” The mental state examination revealed auditory hallucinations and paranoid delusions. He scored 19 on the mini-mental state exam (MMSE). His medical records over the past 2 years indicated visits to the dermatology, ophthalmology, and dentistry clinics. He was diagnosed with chronic gingivitis, pulpitis, and caries into pulp in dentistry clinic. In 2010, the patient presented to ophthalmology clinic with a pigmented lesion in the left lower eyelid which was present since childhood. One year prior to visiting ophthalmology clinic, the patient noticed having additional lesion adjacent to it. No itching over the lesion was present and the patient denied excessive tearing or trauma. He was noticed to have ulceration and occasionally oozing from the lesion. Excision biopsy of the lesions were performed which revealed basal cell carcinoma. His visits to dermatology clinics indicated previous presentations with pitted keratosis involving hands, callosities, and seborrheic dermatitis. On examination, there were numerous palmar pits, red-coloured and measuring about 1-2 mm in diameter (Figure 1). Brain CT scan revealed extensive calcification along falx cerbri and around the cerebellar vermis and few tiny calcifications in the cerebellum bilaterally and in the high parietal regions at the vortex (Figures 2(a) and 2(b)). He had low (20 ng/L) vitamin D level (normal range between 75 and 250 ng/L). Parathyroid hormone level was high at 114.3 ng/L (normal range is 12–88 ng/L). He had high calcium level (2.65 mmol/L), normal phosphorus (0.96 mmol/L), and normal magnesium level. His Chest X-ray was normal, while an X-ray report of cervical spine, done in 2010, revealed loss of cervical lordosis and sclerosis of the endplates with anterior calcification at the level of C5-C6. There was also a mild kyphosis at the level of C4-C5. Posterior facets were reported to have shown sign of degeneration.

## 3. Discussion

The diagnosis of Gorlin-Goltz syndrome requires presence of two major criteria or one major and two minor criteria (Table 1). The major criteria required are multiple (>2) basal cell carcinomas (or one if under 20 years of age), odontogenic keratocysts of the jaws proven by histopathology, 3 or more palmar or plantar pits, bilamellar calcification of the falx cerebri, bifid, fused or markedly splayed ribs, and first-degree relatives with nevoid basal cell carcinoma.

In this reported case, our patient had 3 of the major criteria, namely, calcification of falx cerebri and cerebellum, palmar pits, and basal cell carcinoma which existed before the age of 20. In addition, he had skeletal anomalies, mild cognitive deterioration, and psychotic features, all of which suggest the diagnosis of Gorlin-Goltz syndrome. Our patient also has psychiatric symptoms of labile mood, cognitive impairment, and paranoid delusions which are known to be among the common features of psychotic disorders like schizophrenia or in mental retardation, both are reported in association with Gorling-Goltz syndrome [8]. It may also be explained as a consequence of hyperparathyroidism secondary to vitamin D deficiency with calcium dysregulation. The diagnosis of functional psychotic disorders in this patient is unlikely in presence of organic pathology. In such a diagnosis the psychiatric symptoms may exacerbate with exacerbations of the organic pathology. This explains why the patient had chronic but low level psychotic symptoms which then became more severe during syndrome exacerbations. There was also objective evidence from the clinical symptoms, laboratory, and radiologic investigations to indicate this patient having a systemic physical disorder causing cerebral dysfunction. We therefore, believe that our patient had an acute exacerbation of his chronic physical condition which had resulted in psychiatric manifestations. This is more likely in light of the more than 100 minor features which have been identified in Gorlin-Goltz syndrome [8–15].

We recommend a multidisciplinary approach to assessment and management of these patients with collaboration between services of dermatology, endocrinology, ophthalmology, neurology, and psychiatry. Management of our patient was directed towards his current symptomatic presentation including antipsychotic medications and vitamin D therapy. He showed improvement in his psychiatric symptoms and was discharged from psychiatric unit with further plan of integrating the efforts of a multispecialist team including dermatologist, neurologist, psychiatrist, and odontologist.

## 4. Conclusion

The reported case highlights the importance of considering rare organic etiologies in the differential diagnosis of patients presenting with psychiatric symptoms. Neuroimaging investigations may help to exclude underlying organic pathology which might be the cause of psychiatric symptoms particularly when the presentation is atypical for functional psychiatric disorders in terms of duration, age at onset, and the nature of symptoms in addition to presence of physical symptoms and signs suggesting organic diagnosis which was demonstrated in this reported case of Gorlin-Goltz syndrome. Early diagnosis of underlying organic pathology and comorbid physical conditions in psychiatric patients is of vital importance for better outcome and prevention of complications.

## Figures and Tables

**Table 1:** Diagnostic criteria for Goling-Goltz syndrome.

The major criteria	
(1) More than 2 basal cell carcinomas or one under the age of 20.	
(2) Odontogenic ***keratocysts***.	
(3) Three or more palmar or plantar pits.	
(4) Bilamellar calcification of the falx cerebri.	
(5) Bifid, fused, or splayed ribs.	
(6) First-degree relative with Neavus basal cell carcinomas.	

The minor criteria	
(1) Macrocephaly adjusted for height.	
(2) Frontal bossing, cleft lip/palate, pectus, and syndactyly of digits.	
(3) Sprengel deformity, pectus, and syndactyly of digits.	
(4) Radiology abnormalities: bridging of sella turcica, hemivertebrae, and flame-shaped radiolucencies.	
(5) Ovarian fibroma.	
(6) Medulloblastoma.	

## References

[1] Jawa DS, Sircar K, Somani R, Grover N, Jaidka S, Singh S (2009). Gorlin Gotz syndrome. *Journal of Oral and Maxillofacial Pathology*.

[2] Pandeshwar P, Jayanthi K, Mahesh D (2012). Gorlin-Goltz syndrome: case report of a rare hereditary disorder. *Case Reports in Dentistry*.

[3] Gorlin RJ, Goltz RW (1960). Multiple nevoid basal-cell epithelioma, jaw cysts and bifid rib. A syndrome. *The New England journal of medicine*.

[4] Casaroto AR, Rocha Loures DCN, Moreschi E (2011). Early diagnosis of Gorlin-Goltz syndrome: case report. *Head and Face Medicine*.

[5] Makarious E, Pastalides AD (2009). Intracranial calcifications. *Applied Radiology*.

[6] Carman JS, Wyatt RJ (1979). Use of calcitonin in psychotic agitation or mania. *Archives of General Psychiatry*.

[7] Carman JS, Post RM, Runkle DC (1979). Increased serum calcium and phosphorus with the “switch” into manic or excited psychotic states. *British Journal of Psychiatry*.

[8] de Amezaga AOG, Arregui OG, Nuño SZ, Sagredo AA, Urizar JMA (2008). Gorlin-Goltz syndrome: clinicopathologic aspects. *Medicina Oral, Patologia Oral y Cirugia Bucal*.

[9] Muzio LL, Nocini PF, Savoia A (1999). Nevoid basal cell carcinoma syndrome. Clinical findings in 37 Italian affected individuals. *Clinical Genetics*.

[10] Lo Muzio L (2008). Nevoid basal cell carcinoma syndrome (Gorlin syndrome). *Orphanet Journal of Rare Diseases*.

[11] Zofková I, Kuklík M, Novák Z, Betka J, Dorazilová V (2000). An unusual case of primary hyperparathyroidism in a woman with Gorlin-Goltz syndrome. *Vnitr Lek*.

[12] Vestergaard P, Thomsen SVS (2011). Medical treatment of primary, secondary, and tertiary hyperparathyroidism. *Current Drug Safety*.

[13] Tang JY, Wu A, Linos E (2010). High prevalence of vitamin D deficiency in patients with basal cell nevus syndrome. *Archives of Dermatology*.

[14] Sahota O (2000). Osteoporosis and the role of vitamin D and calcium-vitamin D deficiency, vitamin D insufficiency and vitamin D sufficiency. *Age and Ageing*.

[15] Levenson JL (2006). Psychiatric issues in endocrinology. *Primary Psychiatry*.

